# Fiber-specific white matter alterations in early-stage tremor-dominant Parkinson’s disease

**DOI:** 10.1038/s41531-021-00197-4

**Published:** 2021-06-25

**Authors:** Christina Andica, Koji Kamagata, Yuya Saito, Wataru Uchida, Shohei Fujita, Akifumi Hagiwara, Toshiaki Akashi, Akihiko Wada, Takashi Ogawa, Taku Hatano, Nobutaka Hattori, Shigeki Aoki

**Affiliations:** 1 Department of Radiology, Juntendo University Graduate School of Medicine, Tokyo, Japan; 2 Department of Radiological Sciences, Graduate School of Human Health Sciences, Tokyo Metropolitan University, Tokyo, Japan; 3 Department of Radiology, Graduate School of Medicine, University of Tokyo, Tokyo, Japan; 4 Department of Neurology, Juntendo University Graduate School of Medicine, Tokyo, Japan

**Keywords:** Diagnostic markers, Parkinson's disease

## Abstract

Using a fixel-based analysis (FBA), we assessed the fiber-specific white matter (WM) alterations in nonmedicated patients with early-stage Parkinson’s disease (PD) with tremor-dominant (TD; *n* = 53; mean age, 61.7 ± 8.7 years) and postural instability and gait disorder (PIGD; *n* = 27; mean age, 57.8 ± 8.1 years) motor subtypes and age- and sex-matched healthy controls (HC; *n* = 43; mean age, 61.6 ± 9.2 years) from Parkinson’s Progression Markers Initiative dataset. FBA revealed significantly increased macrostructural fiber cross section and a combination of fiber density and cross section metrics within the corticospinal tract in patients with TD-PD compared with HC. Nonetheless, no significant changes in FBA-derived metrics were found in patients with PIGD-PD compared with HC or patients with TD-PD. Our results may provide evidence of WM neural compensation mechanisms in patients with TD-PD marked by increases in fiber bundle size and the ability to relay information between brain regions.

## Introduction

Parkinson’s disease (PD) is a progressive neurodegenerative disorder, mainly characterized by cardinal motor symptoms, including rigidity, resting tremor, and bradykinesia, as manifestations of selective loss of dopaminergic neurons in the substantia nigra pars compacta and the widespread aggregation of α-synuclein in the form of Lewy neurites and Lewy bodies^1^. Based on predominance of specific motor signs at the time of diagnosis, two main subtypes of Parkinson’s disease (PD) are described: tremor dominant (TD) and postural instability and gait disorder (PIGD)^2^. Discrete patterns of clinical progression have been suggested in TD-PD and PIGD-PD. PIGD-PD is associated with a faster rate of motor decline and weaker response to levodopa treatment^3^, while TD-PD is known to have a better prognosis and slower disease progression^2^. Different clinical presentations and progression of the two PD motor subtypes suggest distinct underlying neural mechanisms. Prior postmortem studies have demonstrated that patients with PIGD-PD had more cell loss in dopaminergic and nondopaminergic neural circuits than the patients with TD-PD^4^. However, while detailed knowledge of the white matter (WM) changes in TD-PD and PIGD-PD, particularly in the early stage, is essential to guide future disease-modifying strategies successfully, to date, the WM structural alterations in TD-PD and PIGD-PD have not been fully elucidated.

Diffusion tensor imaging (DTI) is currently the most widely used approach to estimate changes in WM microstructural integrity in PD^5^. PD has generally been associated with reduced fractional anisotropy (FA)^6^. FA corresponds to the degree of directionality of water diffusion and reflects the white matter integrity^7^. However, a recent study in patients with early-stage TD-PD reported greater FA in WM motor pathways compared with healthy controls (HC) and patients with PIGD-PD, which was interpreted as a result of greater organization of the WM to compensate for PD pathology^8^. However, the interpretation might not be strictly accurate, given the limitations of the tensor model. The inability to resolve multiple fiber orientations in regions of crossing or kissing fibers, which includes up to 90% of the WM voxels in the brain, may contribute to unreliable estimation of DTI measures in these regions^9^. A selective loss of specific fiber directions in crossing-fiber regions may also result in increased FA^10^. Furthermore, FA is influenced by numerous tissue characteristics, including axonal diameter, fiber density, tissue geometry, and degree of myelination;^11^ thus, interpretation of change in such metric must be done with care.

Advances in diffusion modeling techniques allow quantification of WM fiber properties in the presence of complex fiber geometry^9,12^. Fixel-based analysis (FBA) is a framework that facilitates measurement of specific fiber-bundle populations within a voxel or so-called “fixel”^13,14^. In brief, FBA utilizes the fiber orientation distributions (FODs) estimated using constrained spherical deconvolution (CSD) techniques, which enables the measurement of microstructural differences in fiber density (FD), macrostructural differences in fiber bundle cross section (FC), or differences arising from a combination of FD and FC (fiber density and cross section [FDC])^14^. In the developing brain, an increase in fixel metrics might result from increasing axonal diameter or axon count^15,16^, whereas a decrease in those metrics could occur due to axonal loss or atrophy, such as in PD^17–19^.

It remains unknown whether the WM microstructural changes detected in early-stage PD, particularly in TD-PD on DTI (indexed by increases in FA)^8,20,21^, reflect selective neurodegenerative or compensatory mechanisms. Moreover, understanding the PD pathogenesis at the initial stages is crucial for treating these patients more efficiently. In the current cross-sectional study, we applied FBA to evaluate fiber-specific WM alterations in nonmedicated patients with early-stage TD-PD and PIGD-PD using Parkinson’s Progression Markers Initiative (PPMI) dataset. Unlike DTI, we hypothesized that FBA may help in differentiating between selective neural loss and neural compensation, marked by a decrease or increase in fixel metrics, respectively. Further, we also hypothesized that WM neural compensation would be present in patients with TD-PD.

## Results

### Demographics and clinical characteristics

We included 123 participants from the PPMI dataset divided into three groups: 43 HC (25 men; mean age, 61.6 ± 9.2 years), 53 patients with TD-PD (32 men; mean age, 61.7 ± 8.7 years), and 27 patients with PIGD-PD (15 men; mean age, 57.8 ± 8.1 years). As shown in Table 1, the groups were not significantly different in terms of age, sex, and years of education. All subjects were without cognitive decline (Montreal Cognitive Assessment [MoCA] score ≥ 26); however, the MoCA scores were significantly higher in patients with PIGD-PD than in those with TD-PD (*P* = 0.013), while no significant differences were found between HC and either patients with TD-PD or PIGD-PD.

**Table 1:** Demographic characteristics of the participants.

	HC	TD-PD	PIGD-PD	*P*-value			
				HC vs. TD-PD vs. PIGD-PD	HC vs. TD-PD	HC vs. PIGD-PD	TD-PD vs. PIGD-PD
Number of subjects	43	53	27				
Sex (male/female)	25/18	32/21	15/12	0.92^**a**^	N/A	N/A	N/A
Age (years)	61.6 ± 9.2	61.7 ± 8.7	57.8 ± 8.1	0.13^**b**^	N/A	N/A	N/A
Years of education	15.2 ± 2.8	15.1 ± 2.9	15.0 ± 3.2	0.99^**d**^	N/A	N/A	N/A
MoCA	28.4 ± 1.1	28.0 ± 1.3	28.7 ± 1.3	0.030^**d**^	0.11^**e**^	0.18^**e**^	0.013^**e**^
Mean SBR	2.3 ± 0.4	1.3 ± 0.3	1.3 ± 0.4	<0.0001^**b**^	<0.0001^**c**^	<0.0001^**c**^	0.995^**c**^
Dominant side (Rt/Lt)	N/A	28/25	11/16				
Disease duration (months)	N/A	7.3 ± 8.5	5.8 ± 6.3	N/A	N/A	N/A	0.77^d^
Hoehn and Yahr stage	N/A	1.5 ± 0.5	1.6 ± 0.5	N/A	N/A	N/A	0.39^**d**^
MDS-UPDRS-III	0.6 ± 1.5	21.0 ± 7.9	21.7 ± 9.4	<0.0001^**d**^	<0.0001^**e**^	<0.0001^**e**^	0.88^**e**^
Mean progression of motor signs	N/A	6.1 ± 5.7	6.2 ± 4.1	N/A	N/A	N/A	0.57^**e**^

*HC* healthy control, *Lt* left, *MoCA* Montreal Cognitive Assessment, *N/A* not applicable, *PIGD-PD* patients with postural instability and gait disorder Parkinson’s disease, *Rt* right, *TD-PD* patients with tremor-dominant Parkinson’s disease, *MDS-UPDRS-III* Movement Disorder Society-Unified Parkinson’s Disease Rating Scale Part III.

Statistical analyses were performed using ^**a**^chi-square test, ^**b**^one-way analysis of variance (ANOVA) test, ^**c**^Tukey HSD test, ^**d**^Kruskal–Wallis test, and ^**e**^Mann–Whitney *U* test.

As expected, the mean striatal binding ratio (SBR) of patients with TD-PD and PIGD-PD was significantly lower (*P* < 0.0001) than in HC, while there was no difference between patients with TD-PD and PIGD-PD. Although not statistically significant, patients with TD-PD had slightly longer disease duration, but lower Hoehn and Yahr stage, Movement Disorder Society-Unified Parkinson’s Disease Rating Scale (MDS-UPDRS) part III total score, and mean progression of motor signs compared with patients with PIGD-PD.

### Whole-brain FBA

**Fig. 1:**
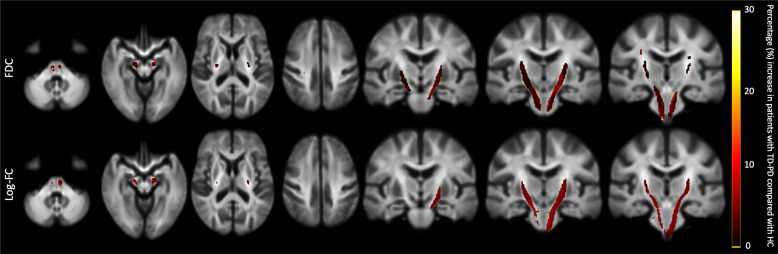
Fiber tract-specific significant FDC and log-FC increases in patients with TD-PD compared to HC. Streamline segments were cropped from the template tractogram to include only streamline points that corresponded with significant fixels (family-wise error-corrected *P* < 0.05). Streamlines were colored by percentage effect increase of log-transformed FC and FDC in the TD-PD group compared with the HC group. FC fiber cross section, FDC fiber density and cross section, HC healthy controls, TD-PD patients with tremor-dominant Parkinson’s disease.

Figure 1 shows the whole brain FBA results for the log-FC and FDC. Compared with HC, patients with TD-PD showed significantly (family-wise error [FWE]-corrected *P*-value < 0.05) higher log-FC and FDC in the bilateral corticospinal tracts (CSTs). No significant differences were observed between other groups (HC vs. PIGD-PD; TD-PD vs. PIGD-PD). There were no WM tracts indicating decreased FD, log-FC, and FDC in patients with PD as opposed to the HC.

### Whole-brain voxel-based analysis (VBA)

No significant changes in FA and mean diffusivity (MD) were demonstrated between any of the tested groups: HC vs. TD-PD, HC vs. PIGD-PD, and TD-PD vs. PIGD-PD.

### Tract-of-interest (TOI) analysis

**Fig. 2:**
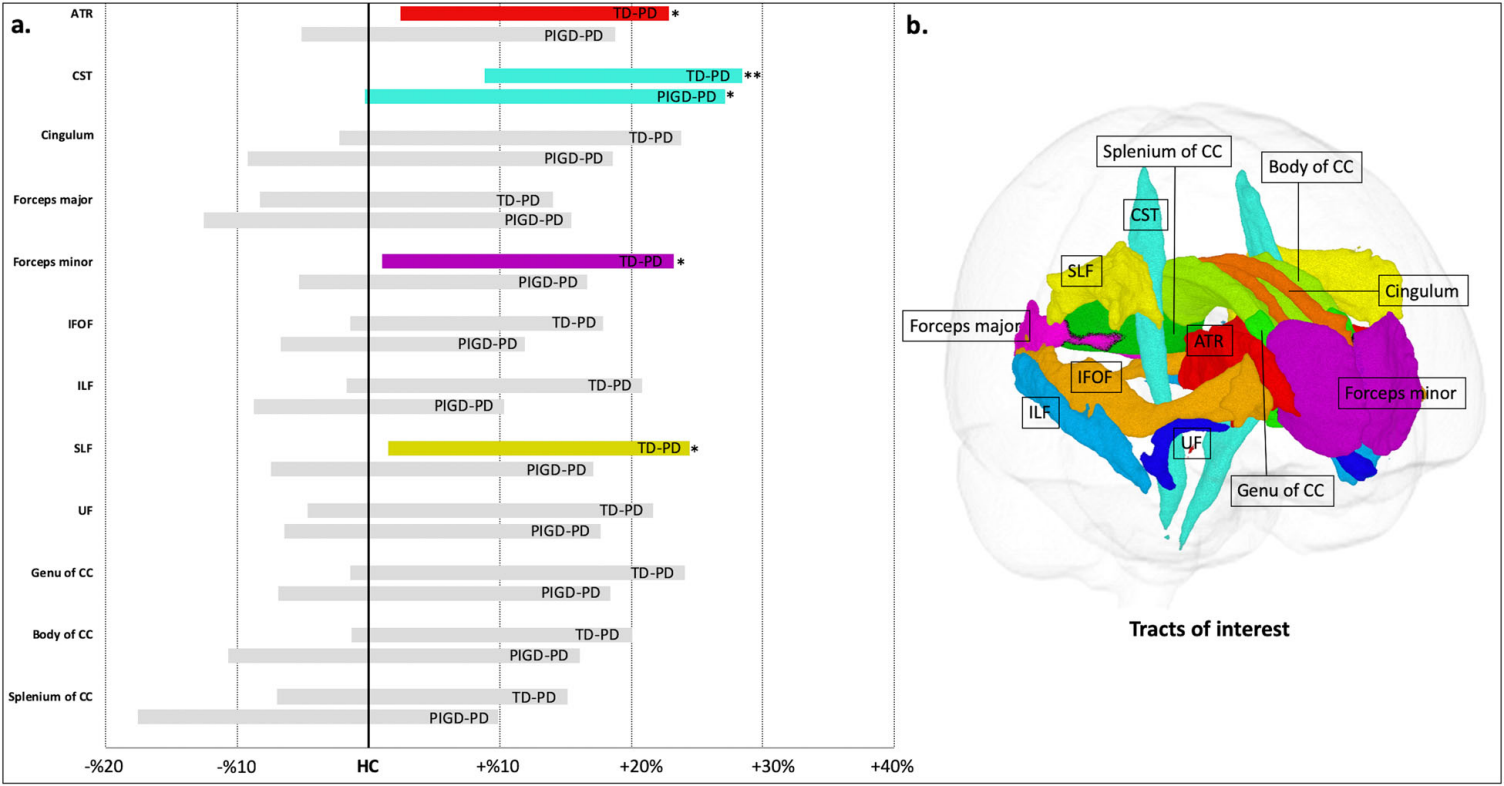
White matter tract of interest analysis. a Increases (displayed in color; *P = 0.0045−0.05, **P < 0.0045) in a combined fiber density and cross section (FDC) metric in patients with TD-PD and PIGD-PD visualized as percentage difference from the HC. **b** Tracts of interest. ATR anterior thalamic radiation, CC corpus callosum, CST corticospinal tract, IFOF inferior fronto-occipital fasciculus, ILF inferior longitudinal fasciculus, PIGD-PD patients with postural instability and gait disorder Parkinson’s disease, SLF superior longitudinal fasciculus, TD-PD patients with tremor-dominant Parkinson’s disease, UF uncinate fasciculus.

Significantly higher mean FDC value was demonstrated in patients with TD-PD compared with HC in the CST (*P* = 0.000036), with large-effect size (Cohen’s *d* = −1.06; Fig. 2 and Table 2). Compared with the HC, there was a trend toward a higher mean FDC value, with medium-effect sizes in the anterior thalamic radiation (ATR; *P* = 0.012, Cohen’s *d* = −0.61), forceps minor (*P* = 0.024, Cohen’s *d* = −0.55), and superior longitudinal fasciculus (SLF; *P* = 0.012, Cohen’s *d* = −0.65) in patients with TD-PD and in the CST in patients with PIGD-PD (*P* = 0.017, Cohen’s *d* = −0.63). There were no significant differences between TD-PD and PIGD-PD. There were no significant correlations between FDC value in patients with TD-PD or PIGD-PD and the disease duration, mean SBR, MDS-UPDRS part III total score, mean progression of motor signs, or MoCA scores.

**Table 2:** Fiber density and cross section values of white matter tracts.

	HC		TD-PD		PIGD-PD		*P*-value				Cohen’s *d*		
	Mean	SD	Mean	SD	Mean	SD	HC vs. TD-PD vs. PIGD-PD	HC vs. TD-PD^a^	HC vs. PIGD-PD^a^	TD-PD vs. PIGD-PD^a^	HC vs. TD-PD	HC vs. PIGD-PD	TD-PD vs. PIGD-PD
ATR	0.49	0.05	0.52	0.05	0.50	0.06	0.014	0.012	0.26	1.00	−0.61	−0.30	0.28
CST	0.74	0.06	0.81	0.07	0.79	0.10	0.000049	0.000036	0.017	0.97	−1.07	−0.65	0.25
Cingulum	0.61	0.06	0.65	0.08	0.63	0.08	0.089	0.084	0.89	1.00	−0.48	−0.21	0.24
Forceps major	0.69	0.08	0.70	0.08	0.70	0.10	0.84	1.00	1.00	1.00	−0.12	−0.06	0.06
Forceps minor	0.46	0.05	0.48	0.05	0.47	0.05	0.028	0.024	0.91	0.66	−0.55	−0.25	0.31
IFOF	0.58	0.06	0.61	0.06	0.59	0.05	0.31	0.096	1.00	0.77	−0.44	−0.14	0.30
ILF	0.54	0.05	0.57	0.06	0.54	0.05	0.051	0.072	1.00	0.26	−0.46	−0.04	0.42
SLF	0.64	0.06	0.68	0.07	0.66	0.08	0.013	0.012	1.00	0.31	−0.63	−0.23	0.36
UF	0.60	0.06	0.63	0.08	0.62	0.07	0.18	0.24	0.59	1.00	−0.37	−0.26	0.12
Genu of CC	0.65	0.09	0.69	0.08	0.67	0.08	0.10	0.10	0.99	1.00	−0.44	−0.22	0.23
Body of CC	0.74	0.08	0.78	0.08	0.75	0.10	0.13	0.14	1.00	0.70	−0.43	−0.11	0.30
Splenium of CC	0.79	0.11	0.81	0.09	0.78	0.11	0.52	1.00	1.00	0.84	−0.16	0.14	0.33

*ATR* anterior thalamic radiation, *CST* corticospinal tract, *HC* healthy controls, *IFOF* inferior fronto-occipital fasciculus, *ILF* inferior longitudinal fasciculus, *PIGD-PD* patients with postural instability and gait disorder Parkinson’s disease, *SD* standard deviation, *SLF* superior longitudinal fasciculus, *TD-PD* patients with tremor-dominant Parkinson’s disease.

^a^Analysis of covariance with Bonferroni-adjusted post hoc tests.

## Discussion

In the current study, FBA was used to measure fiber-specific WM changes in nonmedicated patients with early-stage TD and PIGD motor subtypes of PD. Increased WM integrity was demonstrated in specific fiber bundles of patients with TD-PD compared with HC. These findings may shed light on our understanding of WM changes related to motor subtypes of PD and can be helpful in the development of disease-modifying therapies.

**Fig. 3:**
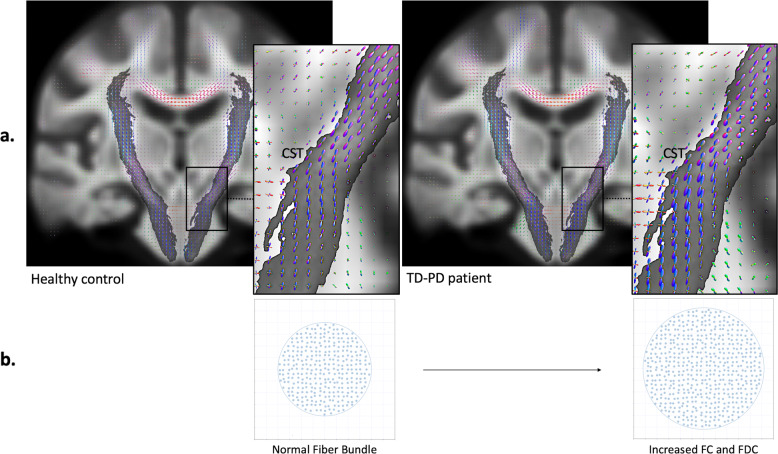
The interpretation of fixel-based metrics changes. **a** Representative images show the enlargement of fiber orientation distributions’ amplitude, which reflects an increase in intra-axonal volume^12^, in the corticospinal tract (CST) in a patient with tremor-dominant Parkinson’s disease (TD-PD) compared with healthy control. **b** The schematic represents a fiber-bundle cross section (blue circles represent axons, and the grid represents imaging voxels). The increase in intra-axonal volume might indicate a fiber-bundle enlargement due to increases in axon number, as indexed by greater fiber cross section (FC) and a combined measure of fiber density and cross section (FDC).

Our findings of changes in the CST and other WM areas, including ATR, forceps minor, and SLF, are consistent with previous work using tensor-derived metrics that showed higher FA in patients with TD-PD compared with HC^8^. Higher log FC and FDC in patients with TD-PD indicate WM macrostructural changes marked by an increase in fiber-bundle size with a higher ability to relay information between the brain regions, respectively^14^. A study evaluating the microstructure of cross-sectioned axons localized to WM tracts within the dorsal striatum of early-stage α-synuclein transgenic mouse brain reported an increased number of axons as a result of axon outgrowth and arborization^22^. Incorporating the histopathological findings, the significant changes in log-FC and FDC in the CST of patients with TD-PD are likely due to the increased number of axons that subsequently enlarged the fiber-bundle size (Fig. 3). Nevertheless, further research, including postmortem histology studies, is required to establish the relationship between physiological tissue changes and fixel metrics.

The observed changes in fixel metrics in the fiber-specific WM tracts provide valuable evidence for neural compensation mechanisms in nonmedicated patients with early-stage TD-PD. A previous study has highlighted the role of dopamine- and non-dopamine-mediated compensatory mechanisms both within and outside the basal ganglia of patients with PD^23^, where one manifestation of the neural compensation might be structural remodeling of neural circuitry^24^. As a consequence, PD has a long premotor or prodromal period during which compensatory mechanisms take place to delay the clinical onset of disabling manifestations, and in the symptomatic period, the neural compensation also has a role in reducing the severity of motor symptoms^24,25^. In line with previous studies^8,26,27^, despite longer disease duration, patients with TD-PD had relatively lower Hoehn and Yahr stages, MDS-UPDRS part III total score, and mean progression of motor signs, as well as higher mean SBR compared with patients with PIGD-PD. Here our results might suggest that the WM neural compensation mechanisms predominantly occurring in the CST could be a key to milder disease severity in TD-PD. The CST is the main WM pathway that conducts motor impulses from the primary motor cortex to the spinal cord, which makes it essential to motor performance in PD^20^. Previous studies showed that the growth in the CST might occur to accommodate the loss of excitability in corticostriatal circuit in the early stage of PD^19,28^. Furthermore, the changes in the CST might be related to the fact that pathological changes in PD progress in an inverse pattern of brain development^29^, considering that most rapid maturation is present in the CST and slowest in the genus of corpus callosum in early childhood^15^. It is also consistent with several previous studies in PD that showed higher FD in the CST with decreased FD in the corpus callosum^19^ and higher FA in the CST with reduced FA within the substantia nigra, corpus callosum, and cingulate gyrus^6^.

A combination of basal ganglia pathology (i.e., pallidal dopamine depletion) and compensation in the cerebello–thalamo–cortical circuit was suggested to lead to tremor in PD, explaining the existence of tremor and a more benign disease course in the same patients^30^. Despite the fact that TD-PD has relatively benign nigrostriatal degeneration compared with PIGD-PD, there is also evidence that resting tremor might emerge as a collateral effect of cerebral mechanisms that compensate for pathophysiological changes producing akinesia^31^. In 1-methyl-4-phenyl-1,2,3,6- tetrahydropyridine primate PD models, tremor usually appears several days after akinesia and rigidity; thus, it was assumed that the appearance of tremor coincides with the time when compensatory mechanisms are presumably being activated^2^. Furthermore, a study with positron emission tomography found higher metabolic activity in M1 that was closely linked to tremor characteristics^32^. The M1, which plays a key role in generating neural impulses that regulate movements, is the target of output from basal ganglia and cerebellum and the site from where a part of the corticospinal descending pathway originates^33^. Thus, it is possible that the compensatory changes in the primary M1 may render this region more susceptible to pathological influences (tremor triggers) from the basal ganglia^30^.

It is also worth noting that the changes in fiber-bundle size are more prominent than changes in density. In contrast, Li et al.^19^ applied the FBA and found higher FD (without significant changes in log FC and FDC) in the CST of treated patients with PD with early (Hoehn and Yahr 1–1.5) and middle (Hoehn and Yahr 2–2.5) stages with unknown disease duration compared with HC. The disagreement between our study and the study from Li et al.^19^ may partly originate from the different sample characteristics and acquisition schemes. Importantly, unlike this study, they did not categorize the PD patients based on the motor subtypes. Moreover, it is known that dopamine replacement therapy is also responsible for neuroplasticity in the WM^34^. These findings might also suggest that WM changes in fiber density and bundle size occur via separate trajectories, which might depend on the timing of the scans. Indeed, the changes of axons might first be detected as a change in FD, and over time further manifest as a difference in log FC^14^. In a longitudinal study of patients with PD with longer disease duration (6.3 ± 5.0 years at baseline) compared with our study, reductions in FD were evident between the first follow-up assessment and baseline, whereas log FC was found to decrease only between the second and first follow-up assessment^17^. Those observations suggest that the first manifestation of WM degeneration in PD might be related to axonal degeneration, subsequently followed by fiber-bundle atrophy^17^. In the same manner, we speculate that neural compensation in PD first increases the FD and causes further enlargement of the fiber bundle.

In contrast to previous studies using PPMI data^8,21^, we found no significant inter-group differences in tensor metrics (FA and MD). This may be due to sample differences (i.e., different inclusion criteria) or differences in the analysis methods used between our and previous studies. The evaluation of DTI metrics is indeed influenced by the analysis techniques^35^. Previous studies utilized a tract-based spatial statistics (TBSS) analysis, the current most commonly used method for VBA of WM diffusion-weighted imaging data^36^. This method applied the skeleton projection step, which intends to reduce the effects of local misregistration. TBSS is generally considered as a whole-brain voxel-based method; however, in fact, only a very small percentage of the WM is investigated since the skeletonization and projection step are based on regions with high FA and therefore, the majority of WM voxels with crossing fibers and low FA are excluded from the analysis. Thus, the projection step is often thought to be less biologically plausible and to have reduced detection accuracy than a whole-brain VBA^37,38^.

Our findings should be interpreted in the context of some limitations. We performed our analysis with single nonzero b-value “single-shell” data only, with limited diffusion-weighting (*b* = 1000 s/mm^2^) rather than multi-shell with higher b-value data that would have better angular resolution and an improved ability to account for partial volume effects^39^. To mitigate this, we utilized the single-shell three-tissue-constrained spherical deconvolution (SS3T-CSD) method, which is capable of isolating the diffusion signal attributable to WM by modeling and removing the contribution from gray matter (GM) and cerebrospinal fluid (CSF)^40^. Further, reverse-phase encoded images were not available for PPMI diffusion-weighted images, and as such, we could not perform correction for magnetic susceptibility-related image distortion that might introduce greater variance in the fixel metrics^41^. It is also important to note, however, that the direct link between fixel metrics and cellular properties has not been robustly validated. Future studies should examine the underlying biological properties measured by fixel metrics. This study only included a small sample size, with a different number of subjects in each group (HC = 43, TD-PD = 53, and PIGD-PD = 27). The proportion of patients with PIGD manifestations in the PPMI cohort is indeed small^42^. These limitations might have limited the power of the statistical analyses and led to false-positive or -negative findings, especially in the PIGD-PD group. A PIGD motor subtype has a higher risk of cognitive decline; however, in this study, MoCA scores were significantly lower in TD-PD than PIGD-PD. We suspected that this resulted from decreased statistical power given that previous PPMI studies evaluating baseline data of patients with TD-PD and PIGD-PD with larger sample sizes showed no significant difference in the MoCA score between the groups^27,42^. Further, a previous PPMI-based DTI study with a smaller sample size (TD-PD, *N* = 52; PIGD-PD, *N* = 13) than our study demonstrated no significant difference in the MoCA score between patients with TD-PD and PIGD-PD^8^. Regardless, all subjects in the current study were without cognitive decline. Intriguingly, in TOI analysis, a nonsignificantly higher mean FDC value was demonstrated in the CST of PIGD-PD group compared with HC. Also, FDC was not significantly different between TD and PIGD groups. We suspect that this was the result of decreased statistical power due to the small sample size. It is also possible that neural compensation occurs in PIGD-PD, but in earlier or shorter times than TD-PD. However, it is important to bear in mind that these results are based solely on cross-sectional data, and so such speculations have to be taken with caution. The limitations of motor clinical scales, which limit the precision of measurement of motor symptoms and impact in early PD^43^, might have prevented detecting differences in clinical motor scores between the TD-PD and PIGD-PD. In Hoehn and Yahr staging, stage II does not necessarily have a more severe motor disability than stage I^44^, while the MDS-UPDRS part III comprises more tremor-related items than posture or gait-related items^45^. Studies evaluating baseline and follow-up (up to 4 years) clinical scores of PD motor subtypes using PPMI data have indeed showed that the differences of Hoehn and Yahr stage or MDS-UPDRS part III total score between TD-PD and PGID-PD were greater with increasing disease duration^8,27^. We also speculated that the narrow range of clinical scores and nonlinear WM changes in the early stage of PD were responsible for the lack of correlation between FDC and clinical measures. Finally, in some patients, tremor severity tends to decrease instead of worsening during disease progression. In this context, the failure of compensatory mechanisms in later stages of the disease leads to gradual disappearance of tremor^30^. Taken together, future longitudinal studies starting from the prodromal to late stages with larger sample sizes are necessary to fully depict the clinical trajectory of fixel-based measure changes in particular fiber pathways and the correlation between fixel-based measures and motor-severity scales in patients with TD-PD and PIGD-PD. Although longitudinal data are also available in the PPMI cohort, it is difficult to directly compare them with the baseline data because most of the patients received treatment by the time of follow-up.

In summary, we implemented FBA and found that patients with TD-PD exhibited growth within the CST. The increase in log FC and FDC in the CST might suggest WM neural compensation mechanisms in early-stage TD-PD marked by enlarged fiber bundle size along with a higher ability to relay information between the brain regions, respectively. Due to this study’s exploratory nature, future studies with histopathological verification are necessary to validate our findings. Further, considering the limitations of this study, the results must be interpreted with caution.

## Methods

### Study participants

The demographic, clinical, and diffusion-weighted imaging data of participants used in this study were downloaded through a standard application process from the PPMI website (http://www.ppmi-info.org/)^46^. The PPMI study was approved by the Institutional Review Board of all participating sites, and written informed consent was obtained from all subjects.

In the current study, we analyzed the baseline data from 80 patients with PD and 43 age- and gender-matched HC. The participants were included in this study if they met the following criteria: (1) they were without cognitive decline, with MoCA score ≥ 26^47^; (2) they had complete data on education years, mean SBR, diffusion-weighted images, and three-dimensional T1-weighted images. Patients with PD were included if they met the following criteria: (1) age at PD onset > 40 years; (2) complete clinical data, including disease duration, Hoehn and Yahr stage, and MDS-UDRS part III total score; (3) at early-stage of PD (Hoehn and Yahr stage ≤ 2 and disease duration < 5 years)^48^, considering that only PD patients with Hoehn and Yahr < 3 had been included in the baseline PPMI cohort^46^; (4) they were not on any PD medications at baseline; (5) confirmed striatal dopamine deficits; (6) the diagnosis of idiopathic PD was maintained after more than two years of follow-up. In the PPMI cohort, the motor subtypes of patients with PD were categorized into TD, intermediate, or PIGD^49^ based on the formula published by Stebbins et al.^45^. Here, patients with PD were included only if they had a predominant type of motor signs (TD-PD, *n* = 53; PIGD-PD, *n* = 27) at the disease onset. The mean progression of motor signs over time was then calculated as the total MDS-UPDRS part III score divided by disease duration in months^26^.

### Diffusion-weighted imaging acquisition

The diffusion-weighted imaging data used in the current study were acquired using a standardized protocol across 11 different sites on 3 Tesla TIM Trio Siemens scanners equipped with a 12-channel head coil. The PPMI diffusion-weighted imaging sequence parameters were obtained using single-shot echo-planar imaging sequence with the following parameters: number of diffusion-encoding directions = 64, b-value = 1000 s/mm^2^, number of nondiffusion (b0) image = 1, repetition time = 900 ms, echo time = 88 ms, matrix size = 116 × 116, slices = 72, flip angle = 90°, voxel resolution = 1.98 × 1.98 mm^2^, and slice thickness = 2.0 mm. More information on the diffusion-weighted imaging acquisition is available online at http://www.ppmi-info.org/.

### Whole-brain FBA

The FBA was implemented using MRtrix3 (http://mrtrix.org) following the recommended pipeline^14^. Preprocessing of diffusion-weighted images included denoising^50^, removal of Gibbs ringing artifacts, eddy-current and motion-induced distortion correction^51^, bias field correction^52^, and upsampling spatial resolution to an isotropic voxel size of 1.3 mm^12^. Following the preprocessing steps, FODs for each subject were calculated using SS3T-CSD approach using a group-averaged response function of each tissue type (WM, GM, and CSF)^53,54^. This step was performed using MRtrix3Tissue (http://3tissue.github.io/), a fork of MRtrix3^55^. In brief, SS3T-CSD allows the estimation of fixed anisotropic single-fiber WM response function and fixed isotropic GM and CSF response functions across the brain using single-shell + *b* = 0 s/mm^2^ data^40,53^. The remaining processing steps included overall image-intensity normalization on subjects’ FOD images to make FOD amplitudes comparable across participants using the median *b* = 0 intensity, generation of a study-specific FOD template using FOD images from all subjects with linear and nonlinear registration^56^, and finally, each subject’s FOD image was subsequently registered to the template^12,56^.

Following the processing steps, three fixel metrics derived from the FBA were calculated as follows:

- FD, a microscopic estimate of the axonal density or packing. In the context of spherical deconvolution^57^ and the apparent fiber-density^12^ frameworks, the amplitude of the FOD along a particular direction is proportional to the magnitude of the diffusion-weighted signal in the perpendicular (radial) plane, hence, to the intra-axonal volume along the corresponding orientation^12^. FD was calculated as the volume of intra-axonal compartment per unit volume of tissue^14^.
- FC, a morphological measure of the macroscopic change in cross-sectional size of fiber bundle. FC is calculated as the extent of distortion in bundle cross section that is required to warp a participant’s FOD into the template image^14^. Prior to statistical analysis, FC was log-transformed (log FC) to ensure zero-centered and normally distributed data. Log FC > 1 or < 1 in the subject compared with the template frame of reference implied a larger or smaller fiber cross section, respectively^14^.
- FDC, a combined measure of FD and FC, calculated as FD multiplied by FC for each fixel. FDC reflects the changes at both microscopic and macroscopic levels, thereby providing sensitivity to any differences related to the capacity of the WM to transmit information^14,16^.

### Whole-brain VBA

FA and MD were calculated using MRtrix3 commands on the same preprocessed images used for FBA. FA and MD maps were subsequently generated in each subject’s space and then transformed to the population template space, by using the same warp generated during the FOD registration step for FBA.

### TOI analysis

We further performed TOI analysis in WM tracts associated with early-stage PD^8,58^, using Johns Hopkins University’s ICBM-DTI-81 WM tractography and labels atlases^59,60^ to guide categorization. The average FDC was computed across each defined tract per participant—namely, ATR, inferior fronto-occipital fasciculus, SLF, inferior longitudinal fasciculus, cingulum, CST, forceps minor, forceps major, genus, body, and splenium of corpus callosum. FDC was chosen for TOI analysis because it is a combined metric of both microstructural and macrostructural changes representing the overall ability to relay information; hence, it is likely to be the most sensitive of the three fixel metrics^14^.

### Volume-based morphometry

Intracranial volume (ICV) of all subjects was obtained using FreeSurfer version 6.0.0 (http://surfer.nmr.mgh.harvard.edu/fswiki) with the recon-all pipeline^61^. We used log-transformed ICV (log-ICV) as a nuisance covariate in whole-brain FBA statistical analysis.

### Statistical analysis

#### Demographic and clinical assessments

All statistical analyses, except for whole-brain FBA and VBA, were performed using IBM SPSS Statistics version 25.0 (IBM Corporation, Armonk, NY, USA). Kolmogorov–Smirnov test was used to assess the normality of the data. The demographic and clinical data were analyzed using the unpaired *t* tests or Mann–Whitney *U* for two groups; one-way analysis of variance (ANOVA; post hoc analysis: Tukey test) or Kruskal–Wallis test were used for comparison of three groups, for normally or non-normally distributed data, respectively, and for continuous variables, while the chi-square test was used for categorical variables. Statistical significance was set at *P* < 0.05.

#### Whole-brain FBA

A general linear model (GLM) framework was utilized to compare FD, log FC, and FDC between (i) HC and TD-PD, (ii) HC and PIGD-PD, and (iii) TD-PD and PIGD-PD, with age, sex, and years of education included as nuisance covariates. To avoid false-positive results, additionally, log-ICV was used as a nuisance covariate for log FC and FDC (but not FD) to remove global effects of brain scaling resulting from the registration to a template^14,62^. Connectivity-based fixel enhancement for statistical inference^13^ with 2 million streamlines from the template tractogram and default smoothing parameters (smoothing = 10-mm full-width at half-maximum, C = 0.5, E = 2, and H = 3)^13^ was used. FWE-corrected *P*-values were then assigned to each fixel using non-parametric permutation testing over 5000 permutations.

#### Whole-brain VBA

Voxel-wise statistical analysis was performed in template space with threshold-free cluster enhancement using default parameters (dh = 0.1, E = 0.5, H = 2)^63^ across the whole brain for the same comparison as in the FBA. An FWE-corrected *P* < 0.05 was considered statistically significant.

#### TOI analysis

Mean FDC value was compared across groups using a one-way analysis of covariance (ANCOVA) followed by pairwise post hoc comparisons (Bonferroni test: HC vs. TD-PD, HC vs. PIGD-PD, and TD-PD vs. PIGD-PD) with age, sex, and years of education as covariates. The effect size was calculated using Cohen’s *d* to evaluate the statistical power of the relationship determined during intergroup comparisons. Effect sizes of 0.2, 0.5, and 0.8 were classified as small, medium, and large, respectively^64^. Correlational analyses of mean tract FDC with the disease duration or mean SBR or MDS-UPDRS part III total score or mean progression of motor signs or MoCA scores of the TD-PD and PIGD-PD were then performed using partial correlation test with age, sex, and years of education included as nuisance covariates. The Bonferroni correction was then used for multiple comparisons of 11 WM tracts with a significance level of *P* < 0.05/11 = 0.0045.

### Reporting summary

Further information on research design is available in the Nature Research Reporting Summary linked to this article.

## Supplementary information

Reporting Summary

## Data Availability

Data used in the preparation of this paper were obtained from the PPMI database (www.ppmi-info.org/data). PPMI data are freely accessible to researchers from the following link: http://www.ppmi-info.org/access-data-specimens/download-data. Access to data collected by PPMI data can be requested by completing the online application (including signing the Data Use Agreement and providing some detailed information). The datasets analyzed during the current study are available from the corresponding author on reasonable request and with permission of PPMI.

## Code availability

All neuroimaging analyses performed in this paper were performed with freely available neuroimaging tools, namely MRtrix3 (http://mrtrix.org/) and FreeSurfer (http://surfer.nmr.mgh.harvard.edu/fswiki). Statistical analyses were performed using IBM SPSS Statistics version 25.0 (IBM Corporation, Armonk, NY, USA).
