# Supplementary material for: Fiber-specific white matter alterations in early-stage tremor-dominant Parkinson’s disease
Source: NPJ Parkinsons Dis. 2021 Jun 25;7:51. doi: 10.1038/s41531-021-00197-4 (PMC8233424; doi:10.1038/s41531-021-00197-4)
Supplement: Supplementary file 1 — Reporting Summary [file 41531_2021_197_MOESM1_ESM.pdf]

nature research

Corresponding author(s): Christina Andica

Last updated by author(s): May 14, 2021

Reporting Summary

Nature Research wishes to improve the reproducibility of the work that we publish. This form provides structure for consistency and transparency in reporting. For further information on Nature Research policies, see our Editorial Policies and the Editorial Policy Checklist.

Statistics

For all statistical analyses, confirm that the following items are present in the figure legend, table legend, main text, or Methods section.

n/a	Confirmed	
☐	☒	The exact sample size (*n*) for each experimental group/condition, given as a discrete number and unit of measurement
☐	☒	A statement on whether measurements were taken from distinct samples or whether the same sample was measured repeatedly
☐	☒	The statistical test(s) used AND whether they are one- or two-sided<br>*Only common tests should be described solely by name; describe more complex techniques in the Methods section.*
☐	☒	A description of all covariates tested
☐	☒	A description of any assumptions or corrections, such as tests of normality and adjustment for multiple comparisons
☐	☒	A full description of the statistical parameters including central tendency (e.g. means) or other basic estimates (e.g. regression coefficient) AND variation (e.g. standard deviation) or associated estimates of uncertainty (e.g. confidence intervals)
☐	☒	For null hypothesis testing, the test statistic (e.g. *F*, *t*, *r*) with confidence intervals, effect sizes, degrees of freedom and *P* value noted<br>*Give P values as exact values whenever suitable.*
☒	☐	For Bayesian analysis, information on the choice of priors and Markov chain Monte Carlo settings
☒	☐	For hierarchical and complex designs, identification of the appropriate level for tests and full reporting of outcomes
☐	☒	Estimates of effect sizes (e.g. Cohen's *d*, Pearson's *r*), indicating how they were calculated

Our web collection on statistics for biologists contains articles on many of the points above.

Software and code

Policy information about availability of computer code

Data collection	The data used in this study were downloaded in July 2020 through a standard application process from the PPMI website (http://www.ppmi-info.org/).
Data analysis	All neuroimaging analyses performed in this manuscript were performed with freely available neuroimaging tools namely MRtrix3 and FreeSurfer. Statistical analyses were performed using IBM SPSS Statistics version 25.0 (IBM Corporation, Armonk, NY, USA).

For manuscripts utilizing custom algorithms or software that are central to the research but not yet described in published literature, software must be made available to editors and reviewers. We strongly encourage code deposition in a community repository (e.g. GitHub). See the Nature Research guidelines for submitting code & software for further information.

Data

Policy information about availability of data

All manuscripts must include a data availability statement. This statement should provide the following information, where applicable:

- Accession codes, unique identifiers, or web links for publicly available datasets
- A list of figures that have associated raw data
- A description of any restrictions on data availability

The data used in this study can be downloaded through a standard application process from the PPMI website (http://www.ppmi-info.org/).

Field-specific reporting

Please select the one below that is the best fit for your research. If you are not sure, read the appropriate sections before making your selection.

☐ Life sciences ☒ Behavioural & social sciences ☐ Ecological, evolutionary & environmental sciences

For a reference copy of the document with all sections, see nature.com/documents/nr-reporting-summary-flat.pdf

Behavioural & social sciences study design

All studies must disclose on these points even when the disclosure is negative.

Study description	The study used retrospective design using the open-access PPMI database.
Research sample	The PPMI baseline data, including healthy controls and patients with Parkinson's disease with tremor dominant or postural instability and gait disorder motor subtypes.
Sampling strategy	No statistical method was used to determine the sample size. We included all participants in the PPMI baseline cohort who met inclusion criteria.
Data collection	Refer to https://www.ppmi-info.org/study-design/research-documents-and-sops/ for documentation describing the recruitment of subjects for the PPMI.
Timing	Refer to https://www.ppmi-info.org/study-design/research-documents-and-sops/ for documentation describing the recruitment of subjects for the PPMI. We downloaded the PPMI data in July 2020.
Data exclusions	The data of participants in the PPMI baseline cohort who did not meet the inclusion criteria were not included in this study.
Non-participation	Refer to https://www.ppmi-info.org/study-design/research-documents-and-sops/ for documentation describing the recruitment of subjects for the PPMI.
Randomization	No randomization was performed.

Reporting for specific materials, systems and methods

We require information from authors about some types of materials, experimental systems and methods used in many studies. Here, indicate whether each material, system or method listed is relevant to your study. If you are not sure if a list item applies to your research, read the appropriate section before selecting a response.

Materials & experimental systems

n/a	Involved in the study	
☒	☐	Antibodies
☒	☐	Eukaryotic cell lines
☒	☐	Palaeontology and archaeology
☒	☐	Animals and other organisms
☐	☒	Human research participants
☒	☐	Clinical data
☒	☐	Dual use research of concern

Methods

n/a	Involved in the study	
☒	☐	ChIP-seq
☒	☐	Flow cytometry
☐	☒	MRI-based neuroimaging

Human research participants

Policy information about studies involving human research participants

Population characteristics	Patients with early-stage Parkinson’s disease with tremor dominant (n = 53; mean age 61.7, SD 8.7 years) and postural instability and gait disorder (n = 27; mean age 57.8, SD 8.1 years) motor subtypes and age- and sex-matched healthy controls (n = 43; mean age 61.6, SD 9.2 years).
Recruitment	Refer to https://www.ppmi-info.org/study-design/research-documents-and-sops/ for documentation describing the recruitment of subjects for the PPMI.
Ethics oversight	The PPMI study was approved by the Institutional Review Board of all participating sites and written informed consent was obtained from all subjects.

Note that full information on the approval of the study protocol must also be provided in the manuscript.

Magnetic resonance imaging

Experimental design

Design type: No task was run during scanning as all MRI scans used were diffusion images.

Design specifications: See Acquisition below.

Behavioral performance measures: No behavioral measures were collected during scanning.

Acquisition

Imaging type(s): Diffusion MRI

Field strength: 3 Tesla

Sequence & imaging parameters: Single shot echo-planar imaging sequence; number of diffusion encoding directions = 64, b-value = 1000 s/mm2, number of non-diffusion (b0) image = 1, repetition time = 900 ms, echo time = 88 ms, matrix size = 116 x 116, slices = 72, flip angle = 90 degree, voxel resolution = 1.98 x 1.98 mm2, and slice thickness = 2.0 mm. More information on the DWI acquisition is available online at http://www.ppmi-info.org/.

Area of acquisition: Whole-brain scans were used.

Diffusion MRI: ☒ Used ☐ Not used

Parameters: Number of diffusion encoding directions = 64, b-value = 1000 s/mm2, number of non-diffusion (b0) image = 1.

Preprocessing

Preprocessing software: MRtrix3Tissue (http://3tissue.github.io/), a fork of MRtrix3, was used to perform fixel-based analysis (FBA) on diffusion-weighted images.

Normalization: Overall image intensity normalization on subjects' fiber orientation distributions (FOD) images was performed to make FOD amplitudes comparable across participants using the median b = 0 intensity.

Normalization template: A study-specific FOD template was generated using FOD images from all subjects with linear and non-linear registration.

Noise and artifact removal: Preprocessing of diffusion-weighted images included denoising, removal of Gibbs ringing artifacts, eddy-current and motion-induced distortion correction, and bias field correction.

Volume censoring: No volume censoring was performed.

Statistical modeling & inference

Model type and settings: Fiber density (FD), fiber cross-sectional (FC), and fiber density and cross-sectional (FDC) of each subject were obtained using FBA.

Effect(s) tested: Group differences of FD, log-FC, and FDC were assessed in this study.

Specify type of analysis: ☐ Whole brain ☐ ROI-based ☒ Both

Anatomical location(s): Tract-of-interest (TOI) analysis was conducted using Johns Hopkins University's ICBM-DTI-81 WM tractography and labels atlases.

Statistic type for inference (See Eklund et al. 2016): Whole-brain FBA: A general linear model (GLM) framework was utilized to compare FD, log-FC, and FDC between groups, with age, sex, and years of education included as nuisance covariates. To avoid false positive results, additionally, log-ICV was used as a nuisance covariate for log-FC and FDC (but not FD) to remove global effects of brain scaling resulting from the registration to a template. Connectivity-based fixel enhancement for statistical inference with 2 million streamlines from the template tractogram and default smoothing parameters (smoothing = 10 mm full-width at half-maximum, C = 0.5, E = 2, H = 3) was used.
TOI analysis: Mean FDC value was compared across groups using a one-way analysis of covariance (ANCOVA) followed by pairwise post hoc Bonferroni comparisons, with age, sex, and years of education as covariates.

Correction: Whole-brain FBA: Family-wise error-corrected P-values were then assigned to each fixel using non-parametric permutation testing over 5000 permutations.
TOI analysis: The Bonferroni correction was used for multiple comparisons of 11 white matter tracts with a significance level of $P < 0.05/11 = 0.0045$.

Models & analysis

n/a	Involved in the study
☒	☐ Functional and/or effective connectivity
☒	☐ Graph analysis
☒	☐ Multivariate modeling or predictive analysis
